# Supplementary figures and images for: Unsupervised Cryo-EM Data Clustering through Adaptively Constrained K-Means Algorithm
Source: PLoS One. 2016 Dec 13;11(12):e0167765. doi: 10.1371/journal.pone.0167765 (PMC5154524; doi:10.1371/journal.pone.0167765)

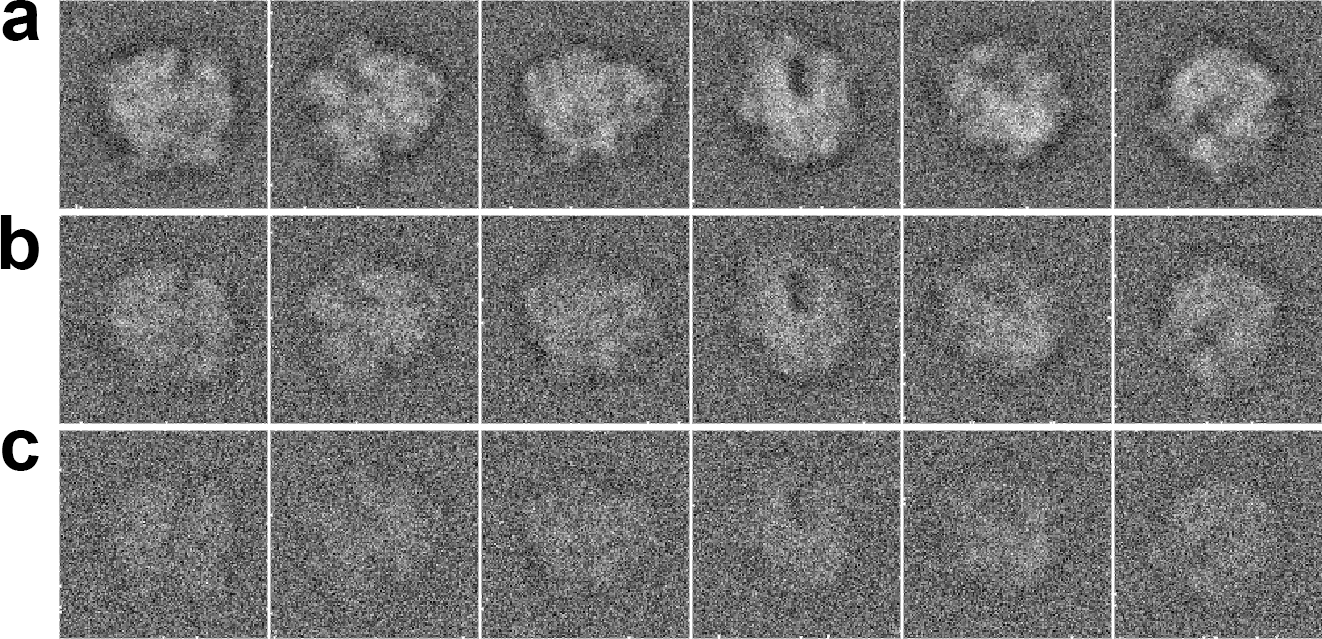

Supplement: S1 Fig — For each noise level, six orientations are shown. (a) SNR = 1/3. (b) SNR = 1/10. (c) SNR = 1/30. (TIF) [file pone.0167765.s001.tif]

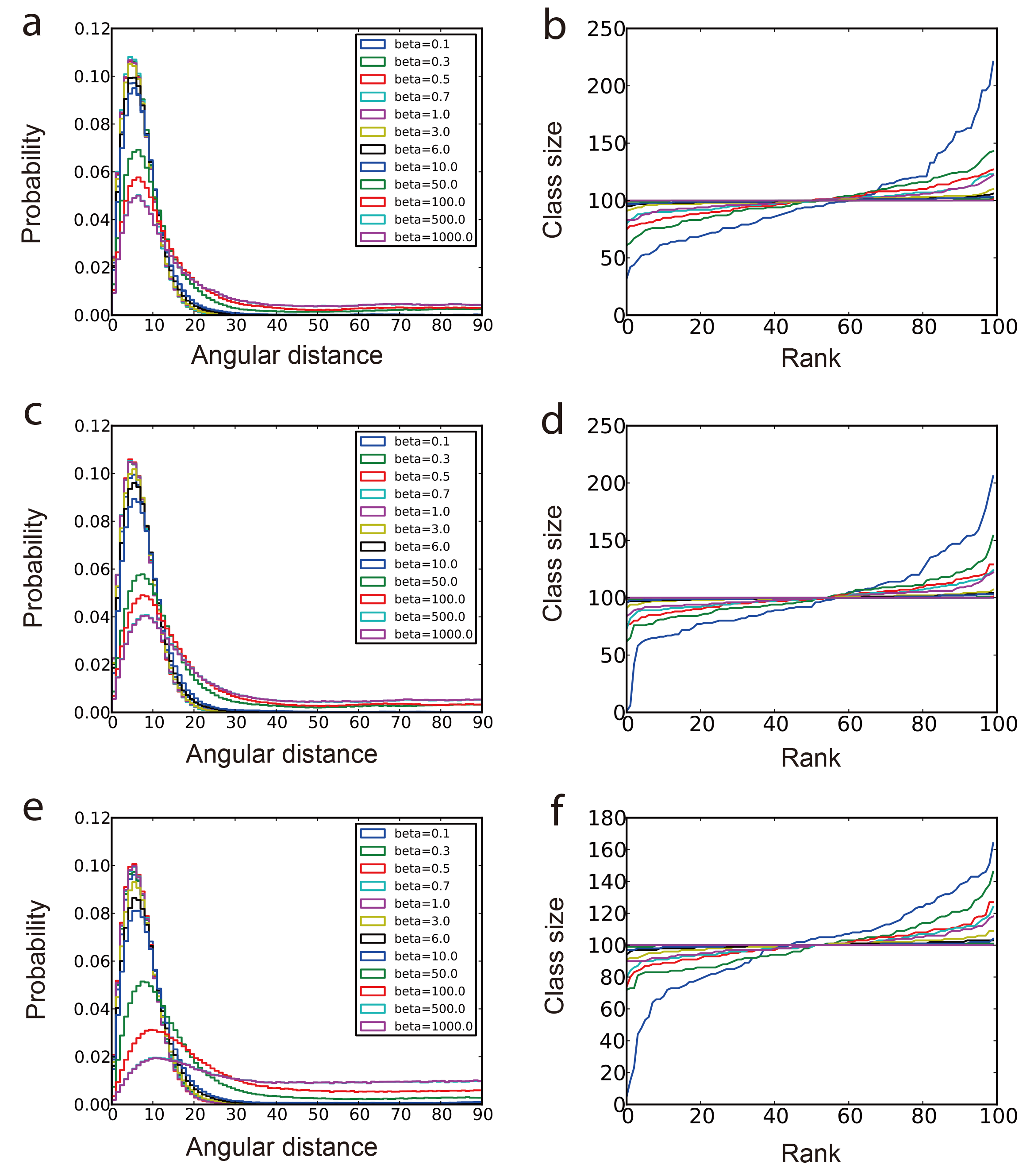

Supplement: S2 Fig — The first column (panels a, c and e) is the normalized histogram of angular distances. The second column (panels b, d and f) is the size of classes which is arranged in ascend order. The most balanced classification has a horizontal line in this plot. The experiments are conducted by the MRA approach in SPARX. (a) and (b) SNR = 1/3. (c) and (d) SNR = 1/10. (e) and (f) SNR = 1/30. (TIF) [file pone.0167765.s002.tif]

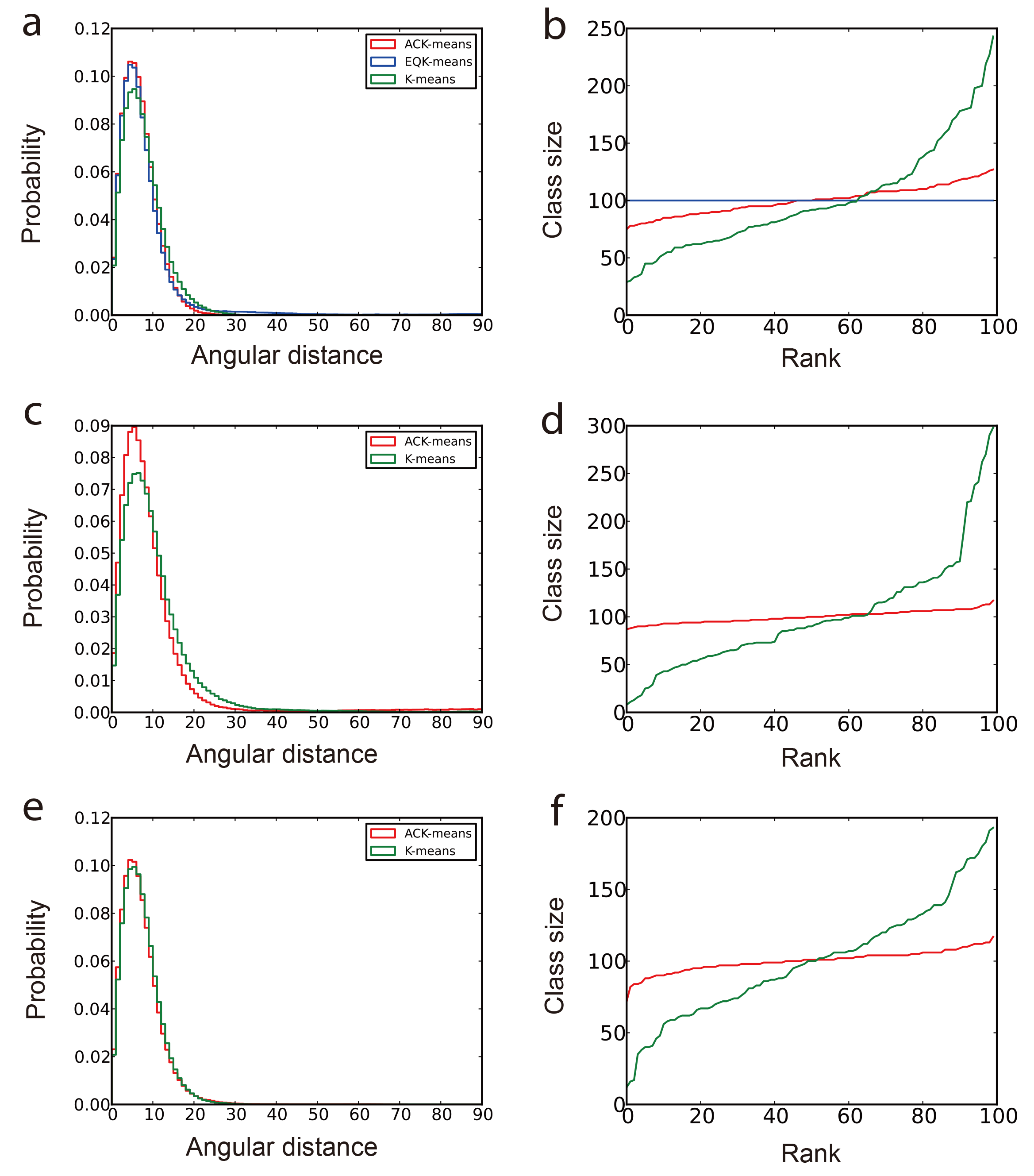

Supplement: S3 Fig — The first column (panels a, c and e) is the normalized histogram of angular distances. The second column (panels b, d and f) is size of classes which is arranged in ascend order. The most balanced classification has a horizontal line in this plot. (a) and (b) are for experiments using different clustering algorithms in the MRA approach under SPARX. (c) and (d) are for experiments using different clustering algorithms in the MRA/MSA approach under EMAN2. (e) and (f) are for experiments using different clustering algorithms in the RFA approach under SPIDER. In all graphs, red curves present the results from the ACK-means algorithm. (TIF) [file pone.0167765.s003.tif]

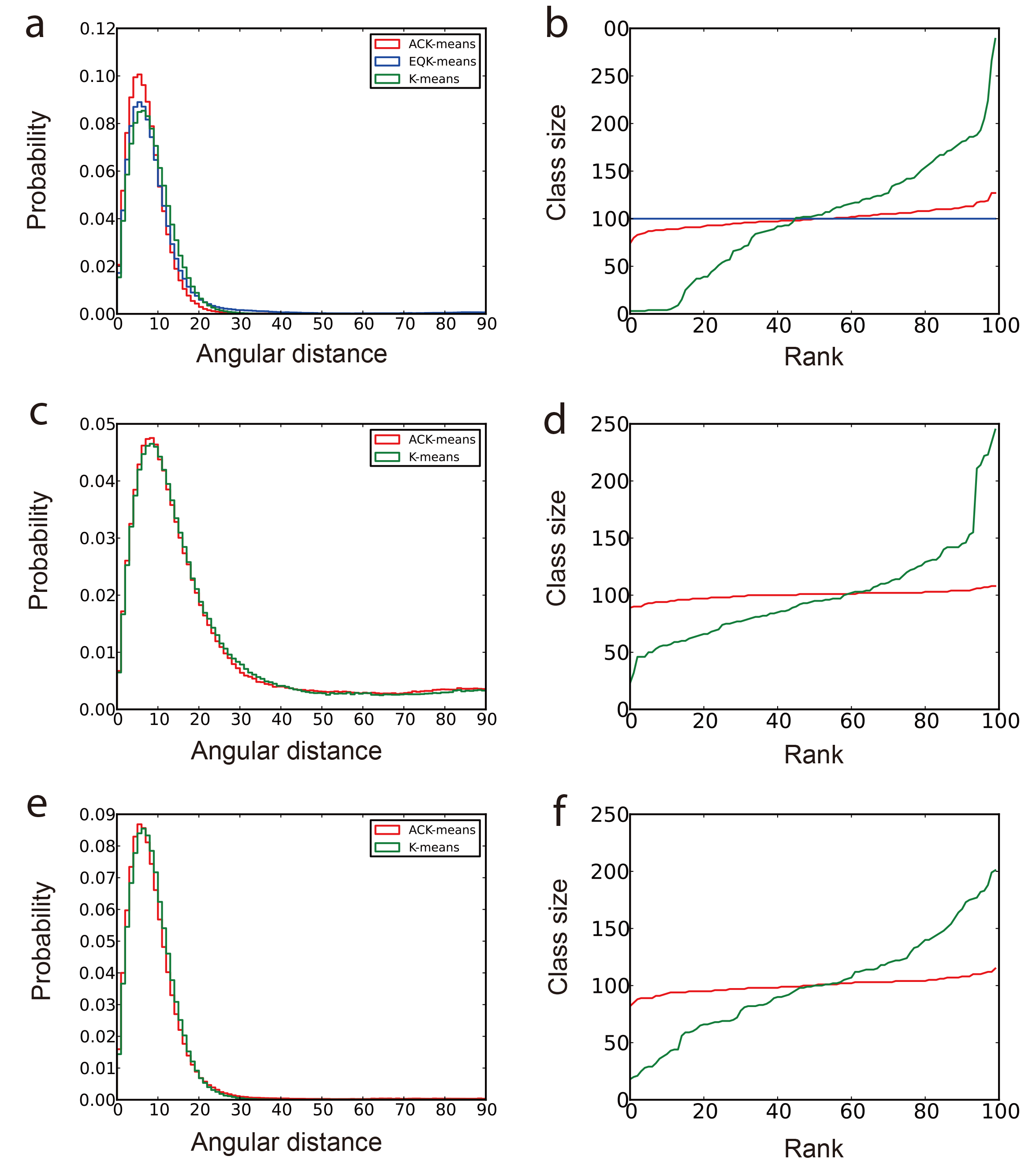

Supplement: S4 Fig — The first column (panels a, c and e) is the normalized histogram of angular distances. The second column (panels b, d and f) is size of classes which is arranged in ascend order. The most balanced classification has a horizontal line in this plot. (a) and (b) are for experiments using different clustering algorithms in the MRA approach under SPARX. (c) and (d) are for experiments using different clustering algorithms in the MRA/MSA approach under EMAN2. (e) and (f) are for experiments using different clustering algorithms in the RFA approach under SPIDER. In all graphs, red curves present the results from the ACK-means algorithm. (TIF) [file pone.0167765.s004.tif]

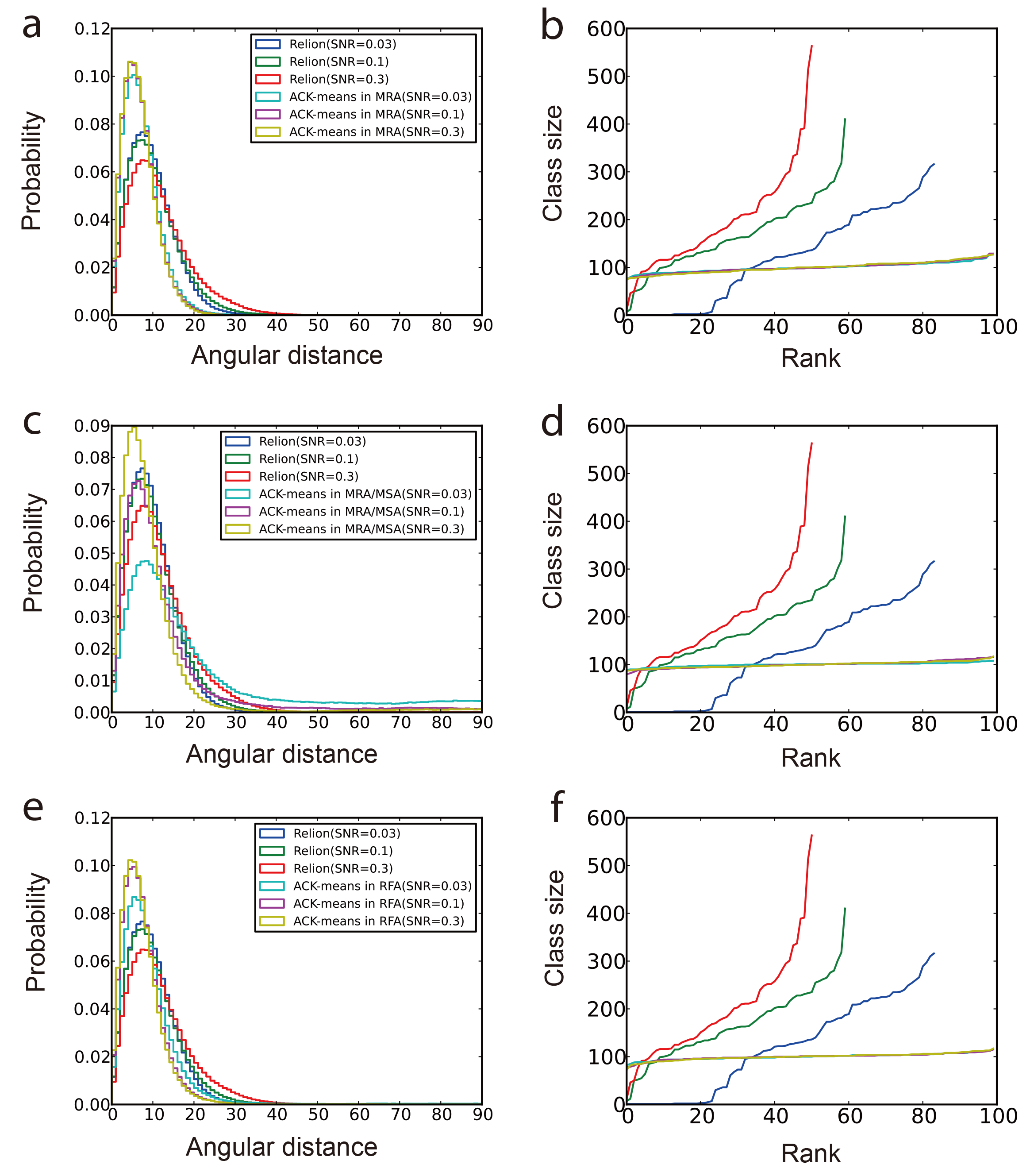

Supplement: S5 Fig — The first column (panels a, c and e) is the normalized histogram of angular distances. The second column (panels b, d and f) is the size of classes which is arranged in ascend order. The most balanced classification has a horizontal line in this plot. (a) and (b) are from ACK-means in MRA and RELION. (c) and (d) are for ACK-means in MRA/MSA and RELION. (e) and (f) are for ACK-means in RFA and RELION. Many classes generated by RELION have no or few particles. (TIF) [file pone.0167765.s005.tif]
